# Ethnic differences in postprandial fatty acid trafficking and utilization between overweight and obese White European and Black African-Caribbean men

**DOI:** 10.1152/ajpendo.00164.2024

**Published:** 2024-07-31

**Authors:** Reuben M. Reed, Fariba Shojaee-Moradie, Gráinne Whelehan, Nicola Jackson, Oliver C. Witard, Margot Umpleby, Barbara A. Fielding, Martin B. Whyte, Louise M. Goff

**Affiliations:** ^1^Department of Nutritional Sciences, Faculty of Life Sciences & Medicine, King’s College London, London, United Kingdom; ^2^Centre for Endocrinology, Diabetes and Research, Royal Surrey NHS Foundation Trust, Guildford, United Kingdom; ^3^Diabetes Research Centre, University of Leicester, Leicester, United Kingdom; ^4^NIHR Leicester Biomedical Research Centre, Leicester, United Kingdom; ^5^Faculty of Health & Medical Sciences, University of Surrey, Guildford, United Kingdom; ^6^Centre for Human & Applied Physiological Sciences, Faculty of Life Sciences & Medicine, King’s College London, London, United Kingdom

**Keywords:** cardiometabolic risk factors, ethnicity, fatty acids, lipid metabolism, stable isotopes

## Abstract

Black African-Caribbean (BAC) populations are at greater risk of cardiometabolic disease than White Europeans (WE), despite exhibiting lower fasting triacylglycerol (TAG) concentrations. However, limited data exist regarding postprandial fatty acid metabolism in BAC populations. This study determined the ethnic differences in postprandial fatty acid metabolism between overweight and obese WE and BAC men. WE [*n* = 10, age 33.3 ± 1.7 yr; body mass index (BMI) = 26.8 (25.8–31.0) kg/m^2^] and BAC [*n* = 9, age 27.9 ± 1.0 yr; BMI = 27.5 (26.0–28.6) kg/m^2^] men consumed two consecutive (at 0 and 300 min) moderate-to-high-fat meals—the first labeled with [U-^13^C]palmitate. The plasma concentration and appearance of meal-derived fatty acids in very-low-density lipoprotein (VLDL)-TAG, chylomicron-TAG, and nonesterified fatty acid (NEFA) were determined over an 8-h postprandial period. Indirect calorimetry with ^13^CO_2_ enrichment determined total and meal-derived fatty acid oxidation rates, and plasma β-hydroxybutyrate (3-OHB) concentration was measured to assess ketogenesis. BAC exhibited lower postprandial TAG [area under the curve (AUC_0–480_) = 671 (563–802) vs. 469 (354–623) mmol/L/min, *P* = 0.022] and VLDL-TAG [AUC_0–480_ = 288 ± 30 vs. 145 ± 27 mmol/L/min, *P* = 0.003] concentrations than WE. The appearance of meal-derived fatty acids in VLDL-TAG was lower in BAC than in WE (AUC_0–480_ = 133 ± 12 vs. 78 ± 13 mmol/L/min, *P* = 0.007). Following the second meal, BAC showed a trend for lower chylomicron-TAG concentration [AUC_300–480_ = 69 (51–93) vs. 43 (28–67) mmol/L/min, *P* = 0.057]. There were no ethnic differences in the appearance of chylomicron-TAG, cumulative fatty acid oxidation, and the NEFA:3-OHB ratio (*P* > 0.05). In conclusion, BAC exhibit lower postprandial TAG concentrations compared with WE men, driven by lower VLDL-TAG concentrations and possibly lower chylomicron-TAG in the late postprandial period. These findings suggest that postprandial fatty acid trafficking may be a less important determinant of cardiometabolic risk in BAC than in WE men.

**NEW & NOTEWORTHY** Postprandial TAG is lower in Black African-Caribbean men than in White European men, and this is likely driven by lower meal-derived VLDL-TAG in Black African-Caribbean men. This observation could suggest that fatty acid trafficking may be a less important determinant of cardiometabolic risk in Black Africans than in White European men.

## Graphical abstract

**Figure:**
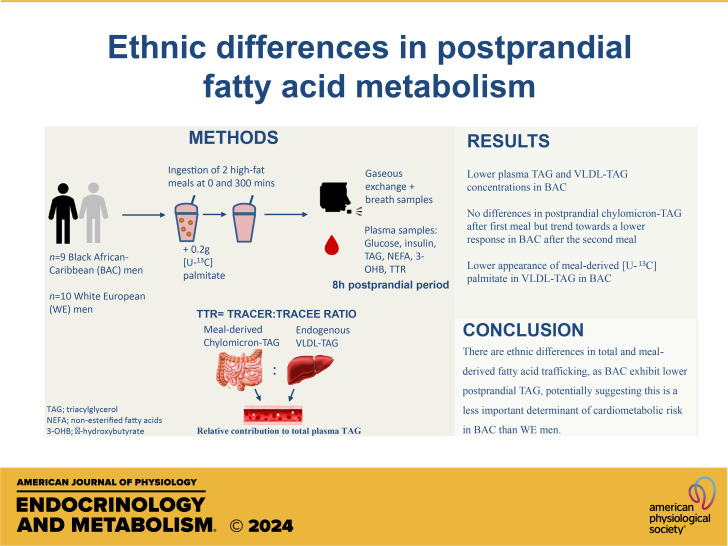

## INTRODUCTION

In the United Kingdom, Black African-Caribbean (BAC) populations are at greater risk of cardiometabolic disease than White Europeans (WE), particularly type 2 diabetes and ischemic stroke (1). Defective lipid metabolism is implicated in the development of cardiometabolic disease, and high fasting triacylglycerol (TAG) concentrations are associated with an increased risk of type 2 diabetes and cardiovascular events (2–4). However, postprandial TAG concentration is reported to be a more powerful predictor of cardiometabolic disease risk (3, 4), which suggests an important role for meal-derived fatty acids in disease pathophysiology.

In the postprandial state, the majority of TAG is transported in TAG-rich lipoproteins, including intestinally derived chylomicrons and hepatically derived very-low-density lipoprotein (VLDL), which carry predominantly meal-derived and endogenous fatty acids, respectively (5). At peripheral tissues, chylomicron-TAG and VLDL-TAG share a common lipolytic pathway via lipoprotein lipase (6), which hydrolyzes TAG, liberating nonesterified fatty acid (NEFA). Following peripheral tissue uptake, fatty acids are either reesterified for storage or undergo β-oxidation for energy generation. In the liver, fatty acids may also be incorporated into VLDL-TAG for export to the circulation or undergo β-oxidation for the production of ketone bodies (7).

Obesity is characterized by elevations in fasting and postprandial TAG concentrations, driven by an overproduction and reduced clearance of TAG-rich lipoproteins (8–10). According to the spillover hypothesis (11, 12), dysfunctional subcutaneous adipose tissue is proposed to increase postprandial TAG (and NEFA) concentrations, driving the deposition of visceral adipose tissue and intrahepatic lipid. The latter may be accentuated by an imbalance between the input and output of fatty acids at the liver, favoring storage over β-oxidation or export (13). The excessive storage of TAG in visceral adipose tissue and the liver are postulated to be primary defects in cardiometabolic disease. Supporting this hypothesis, visceral adipose tissue is more closely associated with cardiometabolic risk factors than subcutaneous adipose tissue (14), and elevations in intrahepatic lipid are understood to drive metabolic dysfunction and the formation of a proatherogenic phenotype (15, 16).

Despite the high risk of cardiometabolic disease in BAC individuals (1), this population exhibits a paradoxically cardioprotective fasting lipid profile, with low fasting TAG and low-density lipoprotein (LDL)-cholesterol concentrations, alongside a higher concentration of high-density lipoprotein (HDL)-cholesterol (17, 18). In addition, BAC typically present with lower visceral adipose tissue (19, 20) and intrahepatic lipid content compared with WE (21). However, a paucity of data exists regarding ethnic comparisons of postprandial fatty acid metabolism between WE and BAC populations, particularly in men, as the majority of studies have been conducted in women (22–25). Conflicting findings include a lower total and incremental TAG response to feeding in young lean BAC compared with WE (26), but recent evidence suggests that BAC exhibit a greater postprandial TAG increment (27). Contrasting observations have also been reported for fatty acid oxidation in men, with studies reporting both similar (28) and lower (29) values in BAC compared with WE. Thus, there remain unanswered questions regarding the ethnic differences in postprandial fatty acid metabolism and whether this may help to explain the increased cardiometabolic risk in BAC men. Accordingly, the overarching aim of this observational study was to investigate ethnic differences in postprandial fatty acid metabolism between WE and BAC men. We utilized gold-standard stable isotope techniques (30) in the first ethnic comparison in overweight and obese WE and BAC men, allowing a comprehensive investigation of total and meal-derived fatty acid metabolism.

## MATERIALS AND METHODS

### Study Design

In an observational study design, postprandial fatty acid trafficking and utilization were compared between WE and BAC men, in response to two consecutive moderate-to-high-fat mixed (macronutrient) meals. Stable isotope methodology was utilized to trace the incorporation of meal-derived fatty acids into plasma TAG, NEFA, chylomicron-TAG, and VLDL-TAG. Ethnic comparisons of total and meal-derived fatty acid oxidation rates and plasma ketogenesis were also conducted. This study was approved by the King’s College London (BDM) research ethics committee (HR/DP-21/22-23409). All participants provided written informed consent. Participant recruitment and data collection were conducted from December 2021 to January 2023.

### Participants

Participants were of self-declared WE (*n* = 10) and BAC (*n* = 10) ethnicity (31), who were overweight or obese but otherwise healthy [eligibility criteria: age 25–40 yr, body mass index (BMI) = 25–40 kg/m^2^, and glycated hemoglobin (HbA1c) < 42 mmol/mol]. Participants were recruited from London by local and social media advertisements and attended an in-person screening visit to confirm eligibility. During this laboratory visit, anthropometric measurements were conducted, and a nonfasted blood sample was collected to measure a full lipid profile, full blood count, HbA1c, and liver and kidney function status. Participants were excluded if they were smokers (or quit within the previous 6 mo), reported medical conditions/medications thought to influence fatty acid metabolism, diagnosed with sickle cell disease (trait permitted), recorded alanine transferase levels >150 IU/L and/or creatinine levels >150 µmol/L, or exhibited dyslipidemia (nonfasted TAG > 3 mmol/L and/or LDL-cholesterol > 5 mmol/L). Body fat percentage/mass was determined by air displacement plethysmography using a BodPod (COSMED, Rome, Italy), utilizing ethnic specific equations of Siri for WE (32) and Schutte for BAC (33).

### Experimental Protocol

Eligible participants attended a single laboratory visit that was conducted in the Metabolic Research Unit at King’s College London, having avoided foods naturally enriched in ^13^C (e.g., popcorn, cornflakes, and corn starch products), alcohol, and strenuous exercise for the preceding 48 h. On the evening before their visit, participants were provided with a standardized meal (containing ∼700 kcal: 45% carbohydrate, 33% fat, and 22% protein), which was consumed before 10:00 PM.

On the day of the visit, participants arrived at the laboratory between 7:00 and 9:00 AM following an overnight fast (>10 h), and a cannula was inserted into an antecubital vein of the forearm. A schematic of the experimental protocol is presented in Supplemental Fig. S1. Two fasting blood samples were obtained (–20 and 0 min) and a fasting breath sample (0 min) was collected in an Exetainer tube (Labco, High Wycombe, UK) using the “drinking straw method” (34). Participants then consumed a moderate-to-high-fat mixed meal (0 min) that contained ∼832 kcal, 52 g fat, and 70 g carbohydrate, with the addition of 0.2 g uniformly labeled ^13^C ([U-^13^C]) palmitate (CK Isotopes, Leicestershire, UK) to label meal-derived fatty acids (Supplemental Table S1; *meal 1*). At 300 min, a second moderate-to-high-fat mixed meal was provided (*meal 2*), which was similar to *meal 1* albeit with less fat (containing ∼652 kcal, 33 g fat, and 70 g carbohydrate; Supplemental Table S1) and no further stable isotope. Participants were asked to consume meals within 10 min. Postprandial blood samples were obtained at 15, 30, 60, 90, 120, 180, 240, 300, 330, 360, 390, 420, and 480 min timepoints. Blood was collected in serum, EDTA, or fluoride oxalate tubes, and serum/plasma were separated by centrifugation. Breath samples were collected at 1-h intervals in Exetainer tubes, and measurements of gaseous exchange, utilizing a GEM indirect calorimeter (GEMNutrition, UK), were conducted to determine whole body CO_2_ production, respiratory exchange ratio, energy expenditure, and substrate utilization rates (assuming negligible protein oxidation) (35) over the 8-h postprandial period.

### Laboratory Analyses

Plasma TAG, NEFA, and glucose concentrations were determined by automated enzymatic colorimetric assays (iLAB 650; Instrumentation Laboratories, Holliston, MA). Serum insulin concentration was determined by immunoassay utilizing chemiluminescent technology and 3-OHB concentration, which is frequently used to represent ketogenesis (36, 37), and was determined by an enzymatic colorimetric assay using an ADVIA Chemistry analyzer (Siemens Healthcare Diagnostics, Camberley, UK).

Density-gradient ultracentrifugation was utilized to separate Svedberg flotation rate (Sf) >400 TAG, approximating chylomicron-TAG, and Sf 20–400 TAG, approximating VLDL-TAG, respectively. Chylomicron-TAG and VLDL-TAG will be used to denote lipoprotein fractions. Samples were overlayed with 1.006 g/mL saline containing 0.1% EDTA (wt/vol) and added to a 50.4 Ti fixed angle rotor within a LE80-K ultracentrifuge. Samples were spun for 20 min at 19,000 rpm to liberate chylomicron-TAG, followed by >16 h at 37,000 rpm to liberate VLDL-TAG. TAG concentration within each fraction was determined by an enzymatic colorimetric assay on a Pentra C400 clinical chemistry analyzer (Horiba ABX, Northampton, UK).

To determine the stable isotope enrichment of plasma TAG, NEFA, chylomicron-TAG, and VLDL-TAG, lipids were extracted and purified by thin layer chromatography. Samples were prepared as fatty acid methyl esters and measured using gas chromatography mass-spectrometry on an Agilent 5975 (Agilent Technologies, Stockport, UK) (38). Breath isotope enrichment was determined by isotope-ratio mass spectrometry (Delta V Advantage, coupled with a Gas Bench II; Thermo Electron, Bremen, Germany) and normalized against the Pee Dee Belemnite international standard (39). Enrichment is presented as follows: [U-^13^C]palmitate concentration, which was determined by inclusion of a triheptadecanoate (TAG) or heptadecanoic acid (NEFA) internal standard; ^13^CO_2_ concentration, which was determined by multiplying the atom percent excess by carbon dioxide production (V̇co_2_) (determined by indirect calorimetry) (39); or the tracer-to-tracee ratio (TTR). Fasting enrichment was subtracted from postprandial samples to account for the natural abundance of ^13^C.

### Calculations and Statistical Analysis

Mean fasting plasma insulin and glucose concentrations were used to calculate the Homeostasis Model Assessment 2 of insulin resistance (HOMA2-IR) (40), using an online calculator (https://www.rdm.ox.ac.uk/about/our-clinical-facilites-and-mrc=units/DTU/software/homa). Total postprandial area under the curve (AUC) was calculated using the trapezoidal rule for the entire postprandial period (AUC_0–480_) and separately in response to the first (AUC_0–300_) and second meal (AUC_300–480_). In the event of missing data, time points were interpolated/extrapolated (<1% of outcome variables). NEFA concentration data were unavailable for one WE participant. Substrate utilization and breath enrichment data were unavailable in another WE participant.

Although no power calculation was conducted, we recruited a similar number of participants as previous studies that investigated meal-derived fatty acid metabolism (37, 41) and a stable isotope study that compared measurements of endogenous fatty acid metabolism between WE and BAC women (25). A post hoc power calculation revealed that for the detection of a difference in [U-^13^C]palmitate AUC_0–480_ of 1 SD, with 90% power and two-sided statistical significance of *P* ≤ 0.05, the following sample sizes are required: 86 per each ethnic group for plasma TAG, 53 per each ethnic group for Sf ≥400 TAG (chylomicron-TAG), and 10 per each ethnic group for Sf 20–400 TAG (VLDL-TAG). Statistical analyses were performed using SPSS (v28; IBM). Ethnic differences in participant characteristics, metabolic variables under fasted conditions (where possible analyzed as the mean of two fasting timepoints), and postprandial AUC of metabolic variables were analyzed using independent samples *t* tests. Data were log transformed where nonnormally distributed or analyzed using Mann–Whitney *U* where a normal distribution could not be obtained. A two-factor repeated-measures ANOVA was conducted for statistical analysis of metabolic variables under postprandial conditions, with time as the within-subject factor and ethnicity as the between-subject factor; pairwise analyses identified ethnic differences at specific time points (Bonferroni-adjusted statistic reported). Data are presented as means ± SE, geometric mean [95% confidence interval (CI)] for log transformed data, or median (IQR) for data analyzed using a Mann–Whitney *U* test.

## RESULTS

### Participant Characteristics

A total of *n* = 30 volunteers were screened for the study: *n* = 9 (6 BAC, 3 WE) did not meet the inclusion criteria, and *n* = 1 (BAC) dropped out before the experimental visit, resulting in *n* = 10 WE and *n* = 10 BAC participants. One BAC participant exhibited extreme results for fasting/postprandial plasma TAG and VLDL-TAG concentrations (>2 SD away from the mean); hence, this participant was considered an outlier and excluded from the analyses. Therefore, data are presented for *n* = 10 WE and *n* = 9 BAC men, unless otherwise stated. Analyses, including the outlier, are reported in Supplemental Material.

The BAC participants were younger and exhibited lower fasting TAG, total cholesterol, and LDL-cholesterol concentrations, compared with WE (Table 1). There were no ethnic differences in BMI, waist circumference, or body fat; in both groups, two participants were obese and the remaining eight (WE) and seven (BA) were overweight. Fasting NEFA, HDL-cholesterol, HbA1c, fasting glucose or insulin concentrations, or HOMA2-IR did not differ between the groups.

**Table 1.** Anthropometric and metabolic characteristics of WE and BAC participants

	WE (*n* = 10)	BAC (*n* = 9)	*P*
Anthropometric characteristics			
Age, yr	33.3 ± 1.7	27.9 ± 1.0	**0.015**
Weight, kg	89.7 ± 3.9	89.2 ± 3.5	0.930
BMI, kg/m^2b^	26.8 (25.8–31.0)	27.5 (26.0–28.6)	0.968
Waist circumference, cm	94 ± 3	90 ± 3	0.460
Body fat, %	22.2 ± 2.7	20.6 ± 2.2	0.661
Fat mass, kg^a^	18.4 (12.8–26.6)	17.3 (12.4–24.1)	0.776
Fat free mass, kg	69.0 ± 1.6	70.5 ± 2.9	0.664
Systolic blood pressure, mmHg	129 ± 3	123 ± 3	0.247
Diastolic blood pressure, mmHg	77 ± 3	71 ± 2	0.112
Metabolic characteristics			
Fasting TAG, mmol/L^b^	0.91 (0.79–1.05)	0.67 (0.51–0.77)	**<0.001**
Fasting NEFA, mmol/L#	0.43 ± 0.07	0.59 ± 0.09	0.180
Total cholesterol, mmol/L	5.05 ± 0.26	4.35 ± 0.21	**0.049**
HDLcholesterol, mmol/L^a^	1.33 (1.15–1.53)	1.43 (1.17–1.75)	0.472
LDLcholesterol, mmol/L	3.52 ± 0.22	2.63 ± 0.24	**0.015**
HbA1c, mmol/mol	32.4 ± 1.0	34.2 ± 1.6	0.337
Fasting glucose, mmol/L	5.7 ± 0.2	5.8 ± 0.2	0.637
Fasting insulin, pmol/L^a^	39.5 (29.2–53.5)	46.7 (40.2–54.3)	0.293
HOMA2-IR^a^	0.8 (0.6–1.0)	0.9 (0.8–1.1)	0.300

Data were analyzed using independent samples *t* tests and are presented as means ± SE. BAC, Black African-Caribbean; BMI, body mass index; HDL, high-density lipoprotein; HOMA2-IR, Homeostasis Model Assessment 2-insulin resistance; LDL, low-density lipoprotein; NEFA, nonesterified fatty acid; TAG, triacylglycerol; WE, White European. Values in bold represent statistically significant results where *P* < 0.05.

^a^Data are presented as geometric mean [95% confidence interval (CI)] for log transformed data;

^b^Data are presented as median (IQR) for data analyzed using Mann–Whitney *U* test.

#Data available in *n* = 9 WE and *n* = 9 BAC.

### Postprandial Metabolite Concentrations

**Figure 1.**
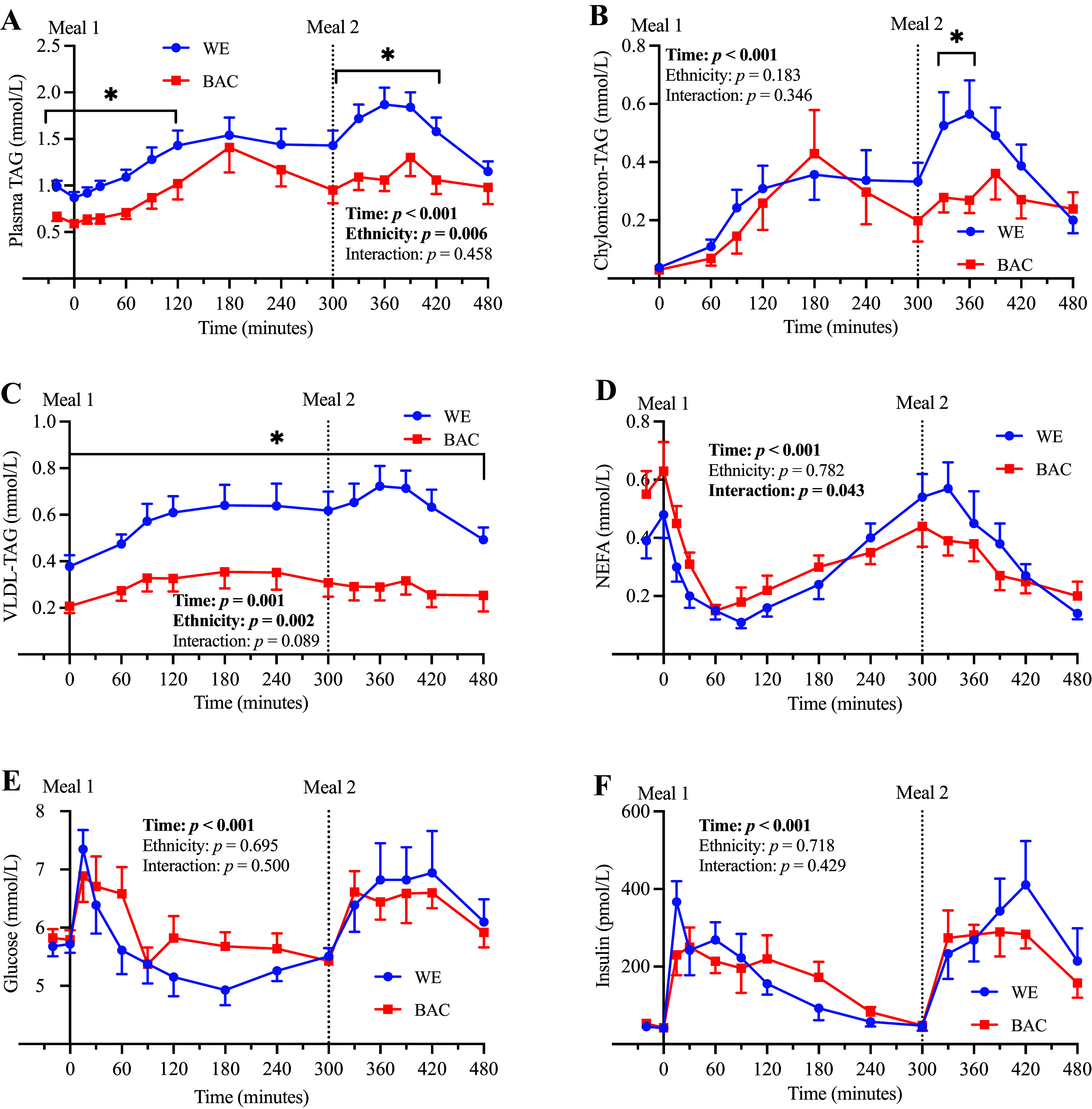
Postprandial metabolite concentrations in WE and BAC men. Postprandial plasma TAG (*A*), chylomicron-TAG (*B*), VLDL-TAG (*C*), NEFA (*D*), glucose (*E*), and insulin (*F*) in WE and BAC men. *Meal 1* and *meal 2* contained approximately 52 and 33 g fat, respectively. Data are presented as means ± SE and were compared using repeated-measures ANOVA with pairwise analyses; *P < 0.05 for effect of ethnicity. BAC, Black African-Caribbean; NEFA, nonesterified fatty acid; TAG, triacylglycerol; VLDL, very-low-density lipoprotein; WE, White European.

Systemic concentrations of plasma TAG, chylomicron-TAG, VLDL-TAG, NEFA, glucose, and insulin, in response to consecutive moderate-to-high-fat mixed meals are presented in Fig. 1 and expressed as AUC in Table 2. BAC exhibited significantly lower plasma TAG concentrations compared with WE at almost all time points (*F* statistic = 9.61, *P* = 0.006; Fig. 1*A*) and a lower total postprandial TAG response, particularly in response to the second meal (AUC; Table 2). The lower plasma TAG in BAC was primarily mediated by a lower VLDL-TAG concentration (*F* statistic = 13.30, *P* = 0.002), which was significantly lower in BAC at all measured timepoints (Fig. 1*C*), including fasting (0 min; WE: 0.41 ± 0.05 vs. 0.20 ± 0.03 mmol/L, *P* = 0.003) and following the first and second meal (AUC; Table 2). There was also a VLDL-TAG time-by-ethnicity interaction (*F* statistic = 2.356, *P* = 0.089). There were no significant ethnic differences in the postprandial chylomicron-TAG concentration or its response to the first meal; however, BAC exhibited significantly lower chylomicron-TAG at 330 and 360 min (Fig. 1*B*), and there was a trend (*P* = 0.057) for a lower second meal chylomicron-TAG response (AUC; Table 2). There were no ethnic differences in postprandial glucose, insulin, or NEFA concentrations between WE and BAC. However, there was a significant time-by-ethnicity interaction for the NEFA response (*F* statistic = 2.60, *P* = 0.043; Fig. 1*D*), but post hoc analyses failed to detect any time point-specific ethnic differences.

**Table 2.** Postprandial plasma lipid concentrations (AUC) in WE and BAC men

	WE (*n* = 10)	BAC (*n* = 9)	*P*	Effect Size
Plasma TAG				
AUC_0–480_^a^, mmol/L/min	671 (563–802)	469 (354–623)	**0.022**	1.153
AUC_0–300_, mmol/L/min	400 ± 37	306 ± 45	0.120	0.752
AUC_300–480_^a^, mmol/L/min	281 (233–340)	183 (139–242)	**0.009**	1.366
Chylomicron-TAG				
AUC_0–480_, mmol/L/min	155 ± 25	119 ± 27	0.345	0.446
AUC_0–300_^a^, mmol/L/min	62 (35–112)	54 (31–93)	0.685	0.189
AUC_300–480_^a^, mmol/L/min	69 (51–93)	43 (28–67)	0.057	0.940
VLDL-TAG				
AUC_0–480_, mmol/L/min	288 ± 30	145 ± 27	**0.003**	1.604
AUC_0–300_, mmol/L/min	173 ± 21	95 ± 17	**0.011**	1.319
AUC_300–480_, mmol/L/min	115 ± 13	51 ± 10	**0.001**	1.801
NEFA#				
AUC_0–480_, mmol/L/min	150 ± 19	145 ± 17	0.834	0.100
AUC_0–300_, mmol/L/min	82 ± 11	90 ± 11	0.616	0.241
AUC_300–480_^a^, mmol/L/min	63 (46–86)	52 (41–66)	0.300	0.505

Data were analyzed using independent samples *t* test and are presented as means ± SE. AUC, area under the curve; BAC, Black African-Caribbean; NEFA, nonesterified fatty acid; TAG, triacylglycerol; VLDL, very-low-density lipoprotein; WE, White European. Values in bold represent statistically significant results where *P* < 0.05.

^a^Data are presented as geometric mean [95% confidence interval (CI)] for log transformed data.

#Data available in *n* = 9 WE and *n* = 9 BAC.

### [U-^13^C]Palmitate Enrichment of Plasma TAG and NEFA

**Figure 2.**
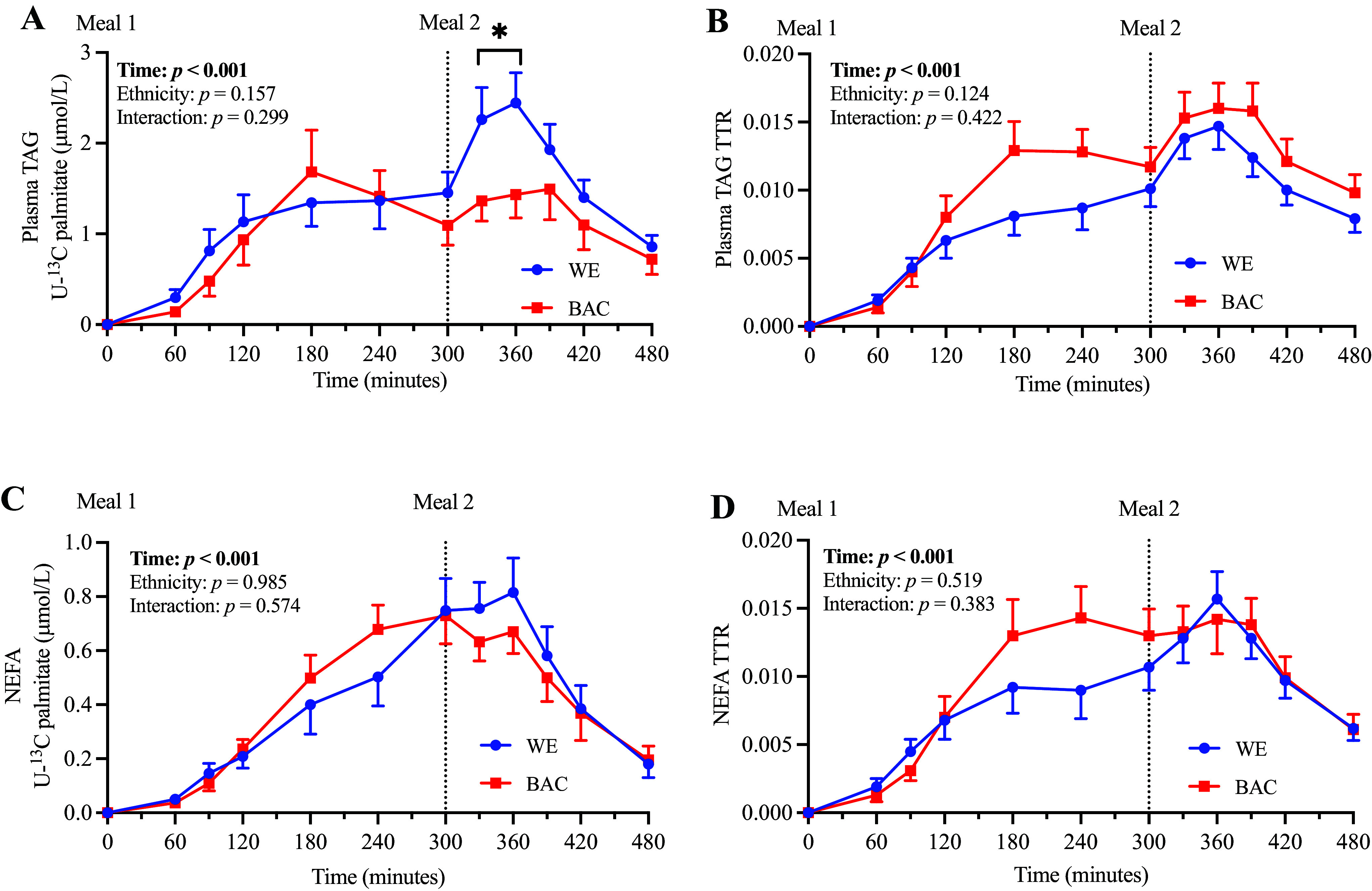
Plasma TAG and NEFA enrichment in WE and BAC men. TAG [U-^13^C]palmitate concentration (*A*), TAG TTR (*B*), NEFA [U-^13^C]palmitate (*C*), and NEFA TTR (*D*) in WE and BAC men. *Meal 1* contained 0.2 g [U-^13^C]palmitate and ∼52 g fat; *meal 2* contained ∼33 g fat. Data are presented as means ± SE and were compared using repeated-measures ANOVA with pairwise analyses; *P < 0.05 for effect of ethnicity. BAC, Black African-Caribbean; NEFA, nonesterified fatty acid; TAG, triacylglycerol; TTR, tracer-to-tracee ratio; [U-^13^C], uniformly labeled ^13^C; WE, White European.

To investigate ethnic differences in meal-derived fatty acid trafficking between WE and BAC men, we measured the meal-derived [U-^13^C]palmitate concentration and TTR of plasma TAG and NEFA (Fig. 2 and expressed as AUC in Table 3). There were no significant ethnic differences in the plasma TAG [U-^13^C]palmitate response between WE and BAC, suggesting a similar appearance of meal-derived fatty acids in plasma TAG over the postprandial period. However, BAC exhibited significantly lower [U-^13^C]palmitate concentration at 330 and 360 min (Fig. 2*A*), suggesting less meal-derived fatty acids from *meal 1* are appearing immediately following the ingestion of *meal 2*. Given that meal-derived fatty acids are transported in chylomicrons, these findings are consistent with a lower concentration of chylomicron-TAG at 330 and 360 min (Fig. 1*B*). There was a trend (*P* = 0.096) for a higher plasma TAG TTR-AUC in BAC compared with WE (AUC; Table 3) that aligned with lower endogenous VLDL-TAG concentrations in BAC (Fig. 1*C*), resulting in a larger relative contribution of meal-derived fatty acids (carried in chylomicrons) to total plasma TAG concentrations. There were no significant ethnic differences in the [U-^13^C]palmitate concentration or TTR of NEFA, suggesting a similar meal-derived NEFA spillover in WE and BAC.

**Table 3.** Plasma TAG and NEFA enrichment (AUC) in WE and BAC men

	WE (*n* = 10)	BAC (*n* = 9)	*P*	Effect Size
Plasma TAG [U-^13^C]palmitate				
AUC_0–480_^a^, µmol/L/min	574 (453–729)	449 (318–634)	0.189	0.628
AUC_0–300_^a^, µmol/L/min	246 (152–397)	241 (157–369)	0.943	0.033
AUC_300–480_, µmol/L/min	310 ± 37	216 ± 38	0.100	0.803
Plasma TAG TTR				
AUC_0–480_	3.9 ± 0.4	4.9 ± 0.4	0.096	0.811
AUC_0–300_	1.8 ± 0.3	2.4 ± 0.3	0.136	0.718
AUC_300–480_	2.1 ± 0.2	2.4 ± 0.2	0.232	0.570
NEFA [U-^13^C]palmitate				
AUC_0–480_^b^, µmol/L/min	167 (157–239)	198 (173–221)	0.400	0.212
AUC_0–300_, µmol/L/min	93 ± 18	108 ± 13	0.501	0.316
AUC_300–480_, µmol/L/min	99 ± 9	88 ± 7	0.338	0.453
NEFA TTR				
AUC_0–480_	4.0 ± 0.4	4.6 ± 0.5	0.376	0.421
AUC_0–300_	1.9 ± 0.4	2.5 ± 0.4	0.308	0.483
AUC_300–480_	2.0 ± 0.2	2.1 ± 0.2	0.888	0.065

Data were analyzed using independent samples *t* tests and are presented as means ± SE. AUC, area under the curve; BAC, Black African-Caribbean; NEFA, nonesterified fatty acid; TAG, triacylglycerol; TTR, tracer-to-tracee ratio; [U-^13^C], uniformly labeled ^13^C; WE, White European.

^a^Data are presented as geometric mean [95% confidence interval (CI)] for log-transformed data;

^b^data are presented as median (IQR) for data analyzed using Mann–Whitney *U* test.

### [U-^13^C]Palmitate Enrichment of Chylomicron-TAG and VLDL-TAG

**Figure 3.**
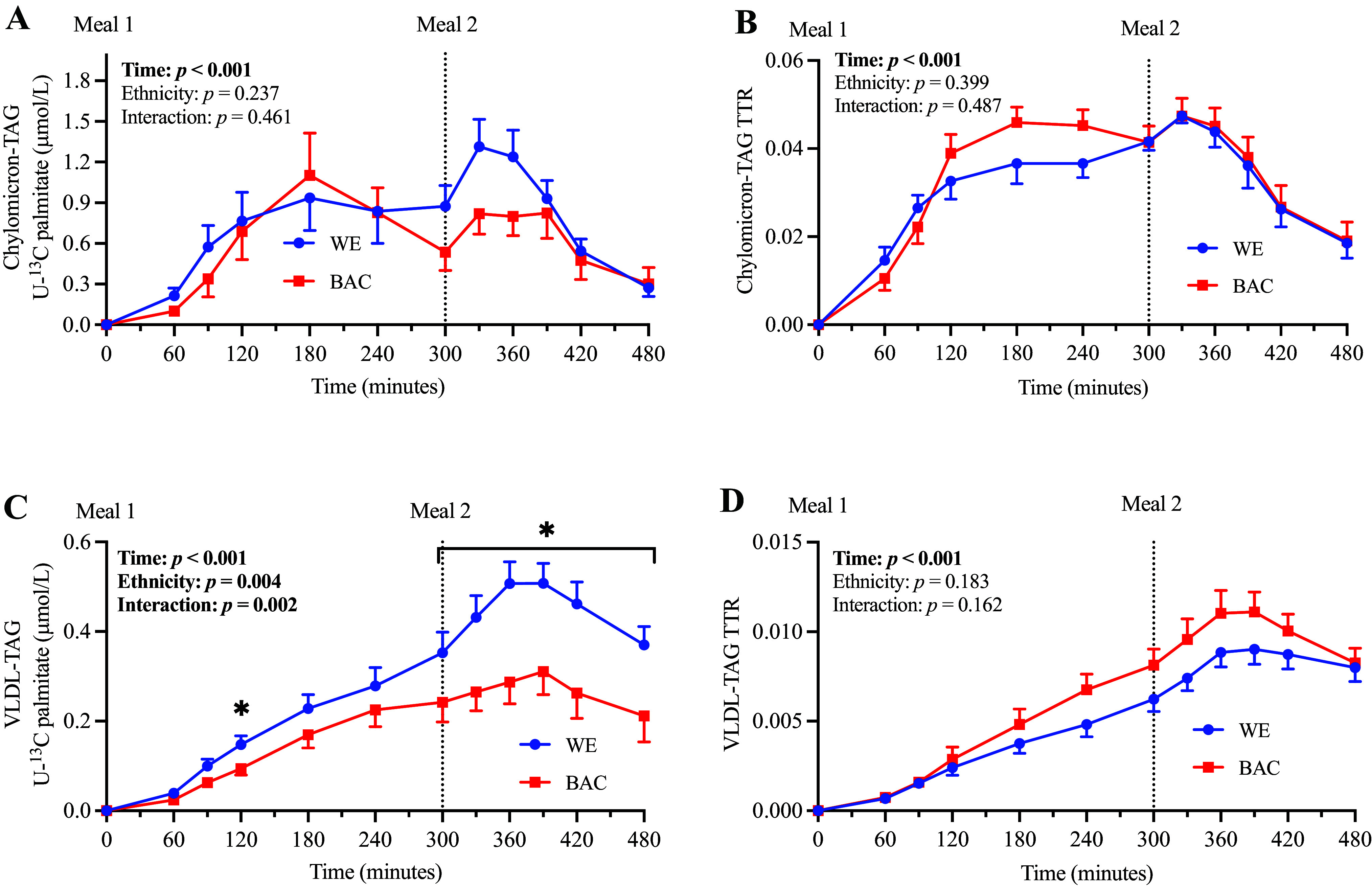
Chylomicron-TAG and VLDL-TAG enrichment in WE and BAC men. Chylomicron-TAG [U-^13^C]palmitate concentration (*A*), chylomicron-TAG TTR (*B*), VLDL-TAG [U-^13^C]palmitate (*C*), and VLDL-TAG TTR (*D*) in WE and BAC men. *Meal 1* contained 0.2 g [U-^13^C]palmitate and ∼52 g fat; *meal 2* contained ∼33 g fat. Data are presented as means ± SE and were compared using repeated-measures ANOVA with pairwise analyses; *P < 0.05 for effect of ethnicity. BAC, Black African-Caribbean; TAG, triacylglycerol; TTR, tracer-to-tracee ratio; [U-^13^C], uniformly labeled ^13^C; VLDL, very-low-density lipoprotein; WE, White European.

To further explore ethnic differences in postprandial meal-derived fatty acid trafficking, density-gradient ultracentrifugation was utilized to separate VLDL-TAG and chylomicron-TAG, before measuring the concentration of meal-derived [U-^13^C]palmitate and TTR of each lipoprotein fraction (Fig. 3 and expressed as AUC in Table 4). The concentration of [U-^13^C]palmitate in VLDL-TAG was lower in BAC compared with WE (main ethnicity effect: *F* statistic = 11.47, *P* = 0.004; time-by-ethnicity interaction, *F* statistic = 5.99, *P* = 0.002; Fig. 3*A*), which was primarily driven by a markedly lower [U-^13^C]palmitate response to the second meal, as well as a trend (*P* = 0.065) for a lower first meal response (AUC; Table 4). This observation suggests a lower appearance of meal-derived fatty acids in VLDL-TAG in BAC versus WE. However, there were no ethnic differences in the VLDL-TAG TTR, suggesting a simultaneous reduction in the appearance of endogenous fatty acids in VLDL-TAG. There were no ethnic differences in chylomicron-TAG [U-^13^C]palmitate concentration or TTR, suggesting the incorporation and secretion of meal-derived fatty acids in chylomicron-TAG are similar in WE and BAC men.

**Table 4.** Chylomicron-TAG and VLDL-TAG enrichment (AUC) in WE and BAC men

	WE (*n* = 10)	BAC (*n* = 9)	*P*	Effect Size
Chylomicron-TAG [U-^13^C]palmitate				
AUC_0–480_^b^, µmol/L/min	335 (198–470)	216 (208–341)	0.447	0.192
AUC_0–300_^a^, µmol/L/min	150 (85–266)	149 (94–234)	0.977	0.013
AUC_300–480_, µmol/L/min	150 ± 19	112 ± 18	0.160	0.674
Chylomicron-TAG TTR				
AUC_0–480_	14.7 ± 0.6	15.9 ± 0.7	0.230	0.573
AUC_0–300_	8.6 ± 0.7	9.6 ± 0.7	0.307	0.484
AUC_300–480_^a^	5.9 (4.9–7.4)	6.1 (5.3–7.3)	0.896	0.079
VLDL-TAG [U-^13^C]palmitate				
AUC_0–480_, µmol/L/min	133 ± 12	78 ± 13	**0.007**	1.416
AUC_0–300_^a^, µmol/L/min	48 (36–66)	33.6 (25–46)	0.065	0.904
AUC_300–480_, µmol/L/min	81 ± 8	42 ± 8	**0.002**	1.676
VLDL-TAG TTR				
AUC_0–480_^a^	2.3 (1.9–2.8)	2.8 (2.2–3.6)	0.176	0.649
AUC_0–300_^a^	0.8 (0.7–1.1)	1.1 (0.8–1.4)	0.159	0.571
AUC_300–480_^a^	1.4 (1.2–1.7)	1.7 (1.3–2.2)	0.231	0.677

Data were analyzed using independent samples *t* tests and are presented as means ± SE. AUC, area under the curve; BAC, Black African-Caribbean; TAG, triacylglycerol; TTR, tracer-to-tracee ratio; [U-^13^C], uniformly labeled ^13^C; VLDL, very-low-density lipoprotein; WE, White European. Values in bold represent statistically significant results where *P* < 0.05.

^a^Data are presented as geometric mean [95% confidence interval (CI)] for log transformed data;

^b^Data are presented as median (IQR) for data analyzed using Mann–Whitney *U* test.

### Ketogenesis and Fatty Acid Oxidation

**Figure 4.**
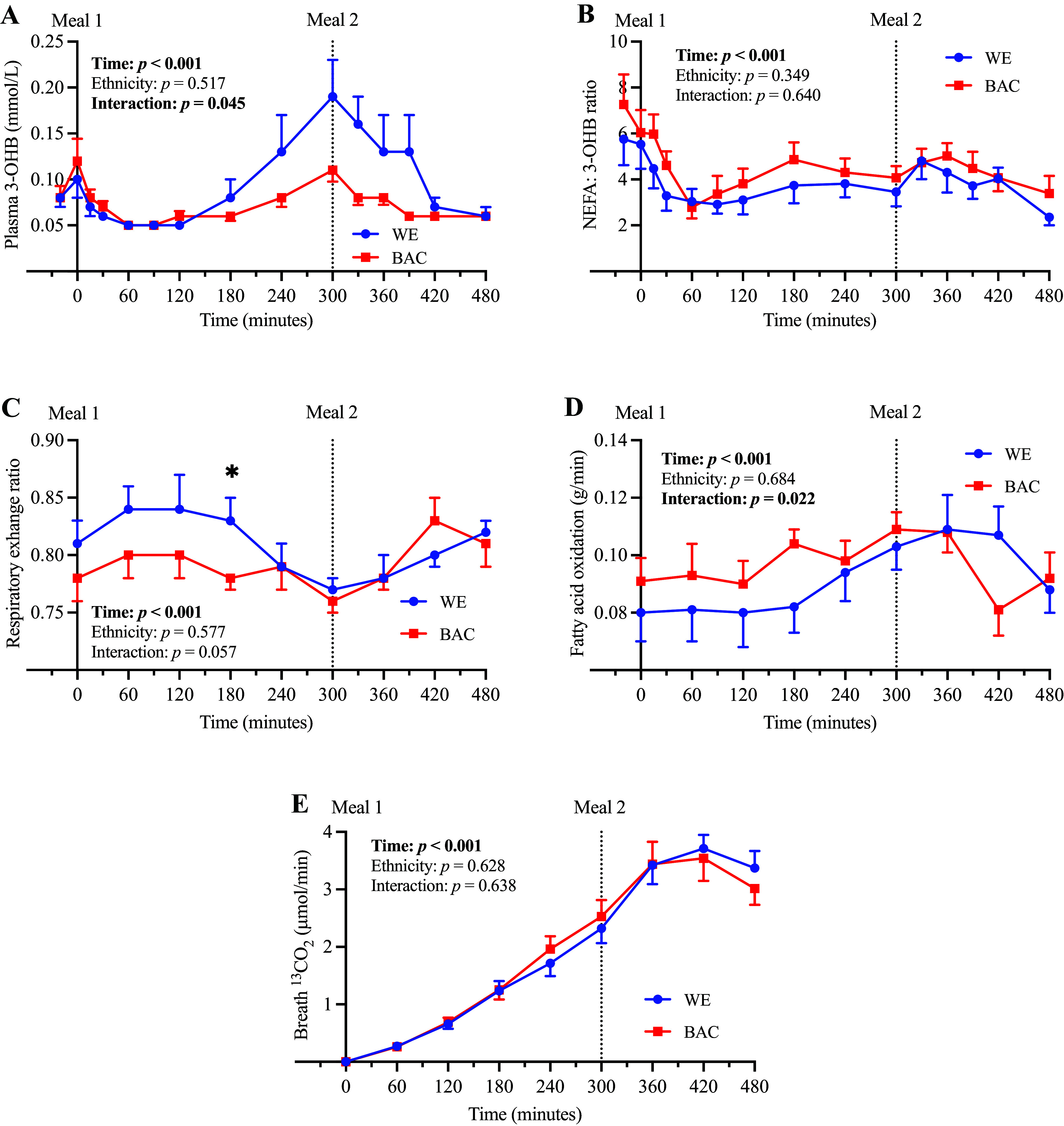
Markers of ketogenesis and fatty acid oxidation in WE and BAC men. Plasma 3-OHB (*A*), NEFA:3-OHB ratio (*B*), respiratory exchange ratio (*C*), fatty acid oxidation (*D*), and breath ^13^CO_2_ concentration (*E*) in WE and BAC men. *Meal 1* contained 0.2 g [U-^13^C]palmitate and ∼52 g fat; *meal 2* contained ∼30 g fat. Data are presented as means ± SE and were compared using repeated-measures ANOVA with pairwise analyses; *P < 0.05 for effect of ethnicity. 3-OHB, β-hydroxybutyrate; BAC, Black African-Caribbean; NEFA, nonesterified fatty acid; [U-^13^C], uniformly labeled ^13^C; WE, White European.

Fatty acids undergo β-oxidation in the liver and may be utilized in ketone body production. Hence, to investigate ethnic differences in hepatic ketogenesis, we measured plasma 3-OHB concentration as the most abundant plasma ketone and calculated the NEFA:3-OHB ratio, as NEFA is the primary substrate for ketone body production (Fig. 4, *A* and *B*, and expressed as AUC in Table 5). Although fasting 3-OHB concentration was similar between ethnicities, BAC exhibited significantly lower 3-OHB in response to the second meal (AUC; Table 5), and there was a significant time-by-ethnicity interaction (*F* statistic = 2.50, *P* = 0.045; Fig. 3*C*); these findings suggest lower hepatic ketogenesis in BAC in the late postprandial period. However, there were no ethnic differences in the NEFA:3-OHB ratio, suggesting ethnic differences in 3-OHB concentration were driven by nonsignificant differences in NEFA concentration (and hepatic delivery), rather than differences in intrahepatic fatty acid partitioning.

**Table 5.** Markers of ketogenesis and fatty acid oxidation (AUC) in WE and BAC men

	WE (*n* = 10)	BAC (*n* = 9)	*P*	Effect Size
3-OHB				
Fasting, mmol/L^a^	0.08 (0.06–0.11)	0.09 (0.06–0.14)	0.548	0.282
AUC_0–480_, mmol/L/min	47.5 ± 7.1	34.1 ± 2.3	0.104	0.788
AUC_0–300_, mmol/L/min^a^	24.3 (17.4–33.8)	20.8 (17.1–25.4)	0.396	0.400
AUC_300–480_, mmol/L/min	20.5 ± 3.2	12.7 ± 0.8	**0.037**	1.052
NEFA:3-OHB ratio#				
Fasting	5.6 ± 1.0	6.7 ± 1.1	0.493	0.331
AUC_0–480_	1,775 ± 216	2,044 ± 231	0.408	0.401
AUC_0–300_	1,058 ± 150	1,271 ± 156	0.339	0.465
AUC_300–480_	717 ± 74	773 ± 86	0.628	0.233
Fasting and cumulative fatty acid oxidation#				
Fasting, g/min	0.08 ± 0.01	0.09 ± 0.01	0.388	0.419
0–480 min, g	17.8 ± 1.6	18.5 ± 1.2	0.708	0.180
0–300 min, g	10.3 ± 1.1	11.6 ± 0.7	0.329	0.474
300–480 min, g	7.5 ± 0.6	6.9 ± 0.5	0.485	0.337
Breath ^13^CO_2_#				
AUC_0–480_^b^, µmol/min/min	801 (754–1,135)	951 (805–1,139)	0.436	0.198
AUC_0–300_, µmol/min/min	303 ± 36	326 ± 37	0.660	0.211
AUC_300–480_, µmol/min/min	599 ± 37	585 ± 61	0.850	0.090

Data were analyzed using independent samples *t* tests and are presented as means ± SE. AUC, area under the curve; BAC, Black African-Caribbean; NEFA, nonesterified fatty acid; WE, White European. Values in bold represent statistically significant results where *P* < 0.05.

^a^Data are presented as geometric mean [95% confidence interval (CI)] for log transformed data;

^b^Data are presented as median (IQR) for data analyzed using Mann–Whitney *U* test.

#Data available in *n* = 9 WE and *n* = 9 BAC.

We also measured the respiratory exchange ratio and total and meal-derived fatty acid oxidation to compare ethnic differences in whole body fatty acid utilization (Fig. 4, *C* and *E*, and expressed as AUC in Table 5). The fasting and postprandial respiratory exchange ratio was similar in WE and BAC men; however, there was a trend for a postprandial time-by-ethnicity interaction (*F* statistic = 2.32, *P* = 0.057), and BAC exhibited a significantly lower respiratory exchange ratio at 180 min (Fig. 4*C*). In line with these findings, there was a significant time-by-ethnicity interaction for fatty acid oxidation (*F* statistic = 2.88, *P* = 0.022; Fig. 4*D*), but fasting and cumulative fatty acid oxidation were similar between WE and BAC (AUC; Table 5). There were no significant ethnic differences in ^13^CO_2_ breath enrichment (AUC; Table 5) or breath TTR (Supplemental Fig. S2), suggesting the oxidation of meal-derived fatty acids was similar in WE and BAC. Energy expenditure and carbohydrate oxidation were also similar between ethnic groups (Supplemental Fig. S3 and Supplemental Table S2).

## DISCUSSION

This study investigated ethnic differences in total and meal-derived fatty acid trafficking and utilization between cohorts of WE and BAC men in response to consecutive moderate-to-high-fat mixed meals. The primary observations were threefold. First, BAC men exhibited lower plasma TAG concentrations over the 8-h postprandial period compared with WE, as mediated by markedly lower fasting and postprandial VLDL-TAG concentrations and potentially lower chylomicron-TAG concentrations following the second meal. Second, the appearance of meal-derived [U-^13^C]palmitate in VLDL-TAG was lower in BAC than in WE men, which likely contributed to the lower VLDL-TAG concentration in BAC, and suggests ethnic differences in the trafficking of meal-derived fatty acids. Finally, although 3-OHB was lower after the second meal in BAC, this difference was abolished following adjustment for its precursor, NEFA, and there were no ethnic differences in total or meal-derived fatty acid oxidation rates. Taken together, these data suggest ethnic differences in total and meal-derived fatty acid trafficking, whereby BAC exhibit lower postprandial TAG than WE, without any ethnic differences in its utilization.

Expanding on previous observations reporting lower fasting TAG in BAC compared with WE populations (17, 18), our data suggest that lower TAG concentrations are maintained in the postprandial state in overweight and obese BAC men. These novel findings are consistent with previous reports of lower postprandial TAG in healthy, young BAC men (26) and women (22, 24), compared with WE counterparts. To investigate potential mechanisms that underpin these ethnic differences in postprandial fatty acid metabolism, we utilized density-gradient ultracentrifugation to isolate TAG-rich lipoproteins approximately corresponding to chylomicron-TAG and VLDL-TAG. The lower fasting and postprandial TAG responses were primarily driven by lower VLDL-TAG concentration in BAC, which were ∼50% lower in BAC compared with WE. Although a lower fasting VLDL-TAG concentration has been reported in BAC men (42), our data are the first to demonstrate lower postprandial VLDL-TAG in overweight and obese BAC compared with WE men.

The concentration of [U-^13^C]palmitate in VLDL-TAG was significantly lower in BAC compared with WE, suggesting a lower export of meal-derived fatty acids as VLDL-TAG in this group. In the postprandial state, meal-derived fatty acids may be incorporated into VLDL-TAG following hepatic uptake of chylomicron remnant-TAG and NEFA derived from the lipolysis of chylomicron-TAG (“NEFA spillover”) (7, 13, 43). NEFA spillover can be quantified by measuring the appearance of meal-derived [U-^13^C]palmitate in plasma NEFA (44). Using this method, we observed no differences in NEFA spillover between WE and BAC, suggesting a similar hepatic exposure of meal-derived fatty acids by this pathway. Taken together, these data suggest a lower uptake of chylomicron remnant-TAG in BAC men, implicating a greater capacity for extrahepatic chylomicron-TAG clearance. Indeed, BAC men are reported to exhibit greater lipoprotein lipase activity (26), and Bower et al. (24) revealed greater subcutaneous adipose tissue expression of lipoprotein lipase in obese BAC compared with WE women. Moreover, given that chylomicron-TAG and VLDL-TAG are both hydrolyzed by lipoprotein lipase (6), lower VLDL-TAG in BAC may reduce the competition for chylomicron-TAG hydrolysis. A trend for lower chylomicron-TAG in the late postprandial period (300–480 min), which was significantly lower at 330 and 360 min, supports the notion that greater TAG clearance is evident in BAC compared with WE.

Conversely, there were no differences in NEFA spillover between BAC and WE men. Given that NEFA spillover is considered a hallmark of effective lipoprotein lipase action (45), these data suggest similar rates of chylomicron-TAG lipolysis and clearance between BAC and WE men. The intrahepatic partitioning of meal-derived fatty acids may also influence the appearance of [U-^13^C]palmitate in VLDL-TAG (7), particularly if storage and/or oxidation (including ketogenesis) are favored. However, BAC populations have been previously shown to exhibit lower intrahepatic lipid than WE (21), and no differences in the NEFA:3-OHB ratio or whole-body meal-derived fatty acid oxidation were observed between ethnicities in this study. Collectively, these findings suggest that mechanisms independent of intrahepatic storage or oxidation account for the lower VLDL-TAG enrichment in BAC compared with WE. Although meal-derived palmitate may also be incorporated into phospholipid and cholesterol ester (41), or undergo elongation or desaturation (37), ethnic differences in these pathways were not investigated in our study. Future studies are warranted to investigate the mechanisms that underpin ethnic differences in the appearance of meal-derived fatty acid in VLDL-TAG.

Despite the lower concentration of [U-^13^C]palmitate in VLDL-TAG in BAC, there were no ethnic differences in the VLDL-TAG TTR, suggesting a parallel reduction in the contribution of meal-derived and endogenous fatty acids to VLDL-TAG in BAC. The primary endogenous substrate for VLDL-TAG synthesis is NEFA of subcutaneous adipose tissue origin (46). Although not measured in this study, similar rates of basal and insulin-stimulated lipolysis have been reported in WE and BAC men (47). Moreover, although we found a significant time-by-ethnicity interaction for NEFA, there were no ethnic differences in fasting or postprandial NEFA concentrations. Collectively, these findings suggest the total supply of adipose tissue-derived NEFA to the liver was similar in WE and BAC. Splanchnic TAG lipolysis (visceral adipose tissue and intrahepatic lipid) may provide additional fatty acids for VLDL-TAG synthesis (43). Visceral adipose tissue is more lipolytically active than subcutaneous adipose tissue (48) and drains directly to the liver via the poral circulation (49). According to the portal theory (11, 49), in addition to providing additional NEFA for VLDL-TAG synthesis, visceral adipose tissue is hypothesized to drive the accumulation of intrahepatic lipid, which is positively correlated with VLDL-TAG output in WE (50). Compared with WE, BAC populations exhibit lower visceral adipose tissue (19, 20) and intrahepatic lipid (21). Such differences may reduce the hydrolysis of splanchnic TAG, reducing the incorporation of endogenous fatty acids into VLDL-TAG in BAC. Alongside the lower incorporation of meal-derived fatty acids, this hypothesis could account for the similar TTR of VLDL-TAG that we observed in BAC and WE groups.

We observed no ethnic differences in the total postprandial response of chylomicron-TAG, or its response to the first meal. Although this study is the first to investigate ethnic differences in direct markers of chylomicron-TAG trafficking, previous studies have measured the incremental TAG response to feeding as an indirect marker of chylomicron-TAG dynamics between ethnicities. Mixed findings have been reported with lower (26) and higher (27) TAG increments observed in BAC compared with WE men and the reasons for discrepant findings between past (26, 27) and present studies are unclear. Interestingly, we observed a trend for lower chylomicron-TAG in BAC following the second meal. Following the consumption of dietary fat, TAG may be stored as intestinal cytoplasmic lipid droplets and subsequently released as chylomicron-TAG following a second feeding stimulus—a phenomenon termed the “second meal effect” (51). Hence, the findings of this study may provide preliminary evidence indicating ethnic differences in this second meal effect. However, despite a tendency toward lower chylomicron-TAG in BAC following the second meal, there were no ethnic differences in chylomicron-TAG [U-^13^C]palmitate enrichment. A similar concentration of meal-derived [U-^13^C]palmitate (from *meal 1*) in chylomicron-TAG, circulating after the second meal, in WE and BAC, therefore does not indicate ethnic differences in the second meal effect/intestinal function. Hence, these findings further support the notion of greater clearance of chylomicron-TAG in BAC, reducing its concentration in the late postprandial period.

In this study, fasting and postprandial fatty acid oxidation rates were similar in BAC and WE men. Although consistent with a previous report (28), this observation does not support the findings of Weyer et al. (29), who reported lower 24-h fatty acid oxidation in BAC men. These discrepant findings may be attributed, at least in part, to ethnic differences in physical activity between studied cohorts. In our study, there were no ethnic differences in meal-derived fatty acid oxidation, suggesting whole body oxidation of total and meal-derived fatty acids was similar in BAC and WE men. In the liver, fatty acids may undergo β-oxidation before being utilized for ketone body production. Interestingly, 3-OHB was significantly lower in BAC than in WE following the second meal; however, with adjustment for NEFA, these ethnic differences were abolished. These findings are consistent with a previous report of similar 3-OHB in BAC and WE when NEFA concentrations were matched during a continuous feeding protocol (52). Therefore, it appears that fatty acids have the same propensity to be shuttled to ketone body production in BAC as WE. Ectopic lipid was not measured in this study. Nonetheless, in accordance with the spillover hypothesis (11, 12), and given that we observed no ethnic differences in fatty acid utilization, it is intuitive that lower fasting and postprandial plasma TAG concentration are contributory mediators of the lower visceral adipose tissue and intrahepatic lipid typically observed in BAC populations (19–21).

These findings are important as they suggest that fatty acid trafficking may be a less important determinant of cardiometabolic disease in BAC than in WE men. However, further work should use a longitudinal design to explore the ethnic differences in the mechanisms linking fatty acid metabolism to ectopic lipid deposition, and to include other measures that impact cardiometabolic disease risk, and that may differ by ethnicity, including composition of the gut microbiome, insulin sensitivity, and insulin clearance. This study has a number of strengths. Postprandial fatty acid metabolism was assessed using gold-standard stable isotope techniques, allowing differentiation of meal-derived fatty acids (30). In addition, postprandial responses were investigated in response to consecutive moderate-to-high-fat mixed meals, which replicates normal feeding behaviors and allows investigation of the second meal effect (51). We also stringently matched BAC and WE men for BMI, body fat percentage, and waist circumference to isolate the effect of ethnicity on postprandial fatty acid metabolism, and BAC exhibited lower fasting TAG and LDL, which is a typical phenotype in this population (17, 18). However, we acknowledge several study limitations. Although density-gradient ultracentrifugation was utilized to approximately separate chylomicron- and VLDL-TAG, this technique does not allow for complete separation of TAG-rich lipoproteins and contamination of VLDL-TAG with small chylomicrons and their remnants remained a possibility (53). However, given the magnitude of the ethnic difference in VLDL-TAG we detected between ethnic groups, we are confident that the lower VLDL-TAG concentrations in BAC compared with WE men were a robust finding. The relatively small sample size (*n* = 19) may have limited the statistical power of the analyses, increasing the likelihood of a type II error, and also possibly leading to some potentially physiologically relevant ethnic differences in fatty acid metabolism being missed. However, robust differences were observed, particularly for VLDL-TAG, which warrant further investigation in larger studies. The BAC men were significantly younger than the WE men, which may have impacted our outcomes; however, our small sample size prevented us from being able to adjust our analyses for this potential confounder. Finally, our study was restricted to a cohort of overweight or obese, but otherwise healthy men. Although lower fasting and postprandial TAG have been reported in BAC women (22, 24), determining ethnic differences in the metabolism of meal-derived fatty acids in women and participants with cardiometabolic disease warrants future investigation.

### Conclusions

This study demonstrated ethnic differences in postprandial TAG trafficking in overweight or obese BAC and WE men. Specifically, the lower fasting and postprandial TAG in BAC than in WE men were mediated by a markedly lower VLDL-TAG concentration. These findings appear somewhat paradoxical given the high prevalence of cardiometabolic disease in BAC populations. Although determining the mechanisms of ethnic differences in the postprandial TAG response is of interest, these findings question the role of fasting and postprandial TAG in the pathophysiology of cardiometabolic disease in BAC men.

## DATA AVAILABILITY

The datasets generated and analyzed during the current study are available from the corresponding author on reasonable request.

## SUPPLEMENTAL MATERIAL

10.25392/leicester.data.26300635 Supplemental Figs. S1–S3, Supplemental Tables S1 and S2, and Supplemental Material: www.doi.org/10.25392/leicester.data.26300635.

## GRANTS

This work was supported by the King’s Medical Research Trust, Joint Research Committee (JRC) PhD Studentship.

## DISCLOSURES

No conflicts of interest, financial or otherwise, are declared by the authors.

## AUTHOR CONTRIBUTIONS

R.M.R., B.A.F., M.B.W., and L.M.G. conceived and designed research; R.M.R., F.S.-M., and N.J. performed experiments; R.M.R., M.U., and B.A.F. analyzed data; R.M.R., B.A.F., M.B.W., and L.M.G. interpreted results of experiments; R.M.R. prepared figures; R.M.R. drafted manuscript; R.M.R., F.S.-M., G.W., N.J., O.C.W., M.U., B.A.F., M.B.W., and L.M.G. edited and revised manuscript; R.M.R., F.S.-M., G.W., N.J., O.C.W., M.U., B.A.F., M.B.W., and L.M.G. approved final version of manuscript.
